# Chloride Diffusion in Concrete Modified with Polyacrylic Superabsorbent Polymer (SAP) Hydrogel—The Influence of the Water-to-Cement Ratio and SAP-Entrained Water

**DOI:** 10.3390/ma14154064

**Published:** 2021-07-21

**Authors:** Maciej Kalinowski, Piotr Woyciechowski

**Affiliations:** Faculty of Civil Engineering, Warsaw University of Technology, 00-637 Warsaw, Poland; p.woyciechowski@il.pw.edu.pl

**Keywords:** superabsorbent polymers, concrete, chloride diffusion, hydrogel, cement, SAP, compressive strength

## Abstract

This paper examines the influence of polyacrylic superabsorbent polymers (SAP) on the properties concerning chloride diffusion in cementitious materials. The conducted study investigated the influence of SAP on chloride diffusion in concretes of the initial w/c = 0.4 (for which the changes in compressive strength due to the SAP presence were negligible). The impact on the diffusivity of concrete of several variables was analyzed: the material characteristics of SAP, additional water added to the concrete to make up for the amount of water stored in the SAP structure, and the method of SAP dosing to the mix (either in a non-saturated form or in a hydrogel form). We found that, in the case of modifying concrete with polyacrylic SAP of a median particle size in dry conditions of 330 µm and without additional water, the coefficient of chloride ion diffusion was reduced to 65% of the reference value. The negative influence (increase) of increasing w/c_tot_ by the amount of water initially entrained by SAP on the chloride diffusivity of concrete was identified. The conducted study indicates the premise of the mechanism of the water release from SAP in cementitious composites.

## 1. Introduction

Current concrete technology incorporates a number of admixtures and additions to improve the properties of cement composites and their durability [1]. The issue of enhancing concrete properties can be approached from different perspectives. Enhancement can be accomplished by changing the chemical composition of the binder through incorporating various additions (fly ash, slag, etc.), modification of the aggregate composition, or admixtures that modify the properties of either the concrete mix or the hardened concrete. However, as cement concrete is produced due to chemical reactions between the concrete mix components, the curing conditions for those reactions also significantly impact the properties of any hardened concrete.

With the introduction of high-performance concretes (HPC) of low water-to-binder ratios, the effect of self-desiccation and the autogenous shrinkage of concrete was identified as a phenomenon that significantly impacted the durability of such composites [2,3]. As conventional curing methods are ineffective in mitigating the autogenous shrinkage of concrete with a low water-to-binder ratio due to limited water migration (due to the low porosity and permeability of HPC), new methods of altering water kinetics within concrete mixtures had to be developed—including internal water curing [4].

Superabsorbent polymers (SAP) can be described as cross-linked hydrogel networks [5]. SAP, depending on the parameters of the water absorption environment, absorb water due to high osmotic pressure caused by the accumulation of ions present within the structure [6]. Absorbing water into SAPs polymer network causes SAP grains to increase their volume, pushing apart ions from each other. With its network stretched out, the osmotic pressure reduces [7].

As SAP absorbs water, the parameters of the water absorption environment also have to be taken into account (its alkalinity and external pressures caused by SAPs volumetric changes) [8]. Thus, to describe the water absorption capacity of any SAP, its maximal water capacity is tested in the distilled water environment in standard conditions (temperature, air pressure conditions, and a lack of hydrostatic pressure) and this is referred to as a reference value [9]. Cement paste is an environment characterized by the presence of different ions influencing the SAP absorption parameters. 

Cement paste can be described as a suspension of physical cement grains in water. Due to this fact, capillary forces within such a system also influence the water transport parameters through forming a pore network [10]. This limits the water absorption capacity of SAP during the absorption stage and prolongs the water desorption process. Due to the combination of those parameters influencing water transport within a cementitious composite, SAP can be used as an internal curing agent [11].

### 1.1. Superabsorbent Polymers (SAP) as Internal Curing Agent—Methods

SAP effectiveness as an internal curing agent is based on its influence on water transport parameters during the hydration of cementitious materials [12]. Internal stresses in the cement matrix due to continuous hydration and self-desiccation of the composite force SAP to release water initially stored in its structure [13]. Due to that mechanism, several parameters of SAP-modified concrete can be altered—the mechanical properties [14,15], degree of hydration [16], autogenous shrinkage [17], parameters of the pore network [18], and others that affect the material’s durability [19]. To control the parameters of water transport in the composite influenced by the presence of SAP, information on the water absorption and water desorption should be gathered from the SAP structure. SAP water absorption and desorption properties are affected by both its chemical composition and its particle size distribution. The influence of the method of SAP introduction into the concrete mix on the water transport within composite should be considered [9].

SAP can be introduced into the concrete mix in two ways. The most common method involves adding non-saturated (‘dry’) SAP to the concrete mix [20]. By doing so, the water absorption phase is limited both in time and volume. After introducing SAP to the mix (usually 0.3–0.6% of the cement mass [20,21]), SAP particles absorb water only for few minutes until reaching their maximal water absorption capacity in a cementitious environment. For polyacrylic SAP, that capacity is usually approximately 10-times lower than the SAP reference water absorption capacity [11]. 

By intentionally lowering the amount of water stored in SAP, the percentage of mixing water intended for internal curing is also limited (to approximately 5–10% [9]). Suppose the mass amount of SAP is increased even further to increase the amount of mixing water absorbed by SAP. In this case, extensive changes to the pore network of the composite can be observed—the formation of defects, and SAP-induced macropores [15,20]. Such severe changes to the pore network negatively influence the mechanical parameters of the materials.

SAP can also be added to the concrete mix in a hydrogel form [9,11]. In this method, SAP is saturated with tap water before its addition to the dry components of the mix. In doing so, it achieves the maximal water absorption capacity in the tap water environment. Knowing that there’s a difference in the volume of water absorbed by polyacrylic SAP in different environments, by introducing fully saturated SAP into the mix, the water desorption process is intensified as it is driven by both internal stresses within a hardening material and the difference in the electrochemical activity of water absorption and desorption environments.

As SAP particles absorb a portion of the mixing water, usually an approach from other internal curing methods, by LWA, for example, is adopted. This involves adding an additional volume of water to the concrete mix to compensate for the water stored in SAP during the mixing phase [9,15]. Due to this fact, three different water-to-cement ratios can be distinguished—the total water-to-binder ratio (w/b_tot_), effective water-to-binder ratio (w_eff_/b), and (w_e_/b)—a ratio of water entrained in SAP to the mass of the binder [11]. 

The amount of water entrained in SAP is experimentally verified either by measuring the amount of water required to keep the consistency of SAP-modified concrete comparable with non-modified variant or based on SAP water absorption tests in an appropriate cementitious environment (in the case of polyacrylic SAPs, the water absorption in cementitious environments is approximately 10-times lower than in a tap water environment) [11,15,20]. 

Although consistency is a vital characteristic of any concrete mix, the addition of extra water to the concrete mix to improve it complicates both the design process of any SAP-related experiments and the understanding of the SAP influence on the properties of cementitious composites. With an introduction of the additional volume of water into the mix, the proportions between all ingredients are subjected to changes (aggregate:cement:water). As SAP absorbs a portion of mixing water during the mixing stage, its volume influences the parameters of the pore network. 

By increasing the overall volume of water in 1 m^3^ of concrete mix, an increase in its porosity is expected, which leads to the deterioration of its mechanical properties and an overall deterioration in its durability [15]. In this and previous research, the authors proposed a different approach to designing SAP-modified concretes—modification by SAP in a hydrogel form, with no additional water added to the system [9,11].

### 1.2. Modification of Concrete with Hydrogels—A New Approach

Different parameters characterize SAP hydrogels and non-saturated SAP. As the water absorption process in SAP is electrochemical in nature, in a cementitious environment, SAP in a non-saturated form tends to agglomerate, resulting in the formation of macropores in the cement matrix, which negatively impacts the mechanical performance of hardened concrete [9,11]. This SAP characteristic is one of the reasons why the amount of SAP added to the concrete mix usually is less than 1% m.c. [20]. 

As the water absorption capacity of polyacrylic SAPs is much lower than in a tap water environment, the increase in the volume of SAP particles while added in a non-saturated form is a fraction of the volume of SAP that is fully saturated in tap water [22,23]. Of course, such an increase in the volume of SAP hydrogel saturated in tap water before mixing with other concrete mix components would negatively impact the homogeneity and overall pore network parameters. 

However, due to the amount of water required to reach the maximal water absorption capacity, the SAP hydrogel is stretched to the maximum allowed by its polymer structure [9]. This fact makes SAP hydrogel susceptible to fragmentation during mixing. In a study performed by the authors, forced change in granulation due to friction with other concrete mix components was investigated. It was found that, due to the mixing conditions, the granulation of tested SAP was changed—the median particle size fell down from approximately 330 µm (non-saturated SAP) to approximately 34 µm (SAP after fragmentation and desorption of water) [9].

While modifying concrete with SAP, several phases of SAP influencing water transport can be distinguished. In the case of dosing non-saturated SAP, a water absorption phase lasts for few minutes after the addition of SAP to the mix, followed by a slow desorption phase caused by internal stresses within the cement matrix (due to hydration autogenous shrinkage, etc.) [9]. In the case of adding SAP in a hydrogel form, the water absorption process takes place outside of the concrete mix, followed by fragmentation of the hydrogel during the mixing stage and intense water desorption from the SAP structure during the first 24 h after forming. 

As SAP is used in concrete technology to desorb water in order to keep the internal humidity at a constant and high level for as long as possible, methods incorporating its use in cementitious composites should consider both the water absorption and desorption characteristics [24]. The parameters of the capillary network of cementitious materials need to be taken into account, as those impact the parameters of water transport within the pore network [24].

The method of modifying concrete with SAP in a hydrogel form is based on the assumption that SAPs water desorption parameters over time are important for achieving an optimal internal curing effect. As the primary goal of internal curing is to evenly distribute and control the water present within the cement matrix in order to mitigate self-desiccation of the cement matrix, one can argue that only the course of water desorption from SAP into cement matrix has a lasting impact on the hydration process of the binder. The SAP absorption capacity in any given environment is limited by the properties of the environment as stated previously. 

In the case of dosing non-saturated SAP to the concrete mix, this deteriorates SAP water absorption approximately 10 times if compared to the absorption capacity in a tap water environment [23]. By changing the environment of SAP water absorption to tap water and then placing the obtained hydrogel in the concrete mix, the water transport between SAP and the cement matrix is reversed [9]. From the moment of introducing hydrogel to the mix, water from SAP is released to the cement mix until SAP reaches its water absorption capacity in a cementitious environment. 

However, as the desorption process occurs in a cementitious environment, due to capillary pressures in the cement matrix and fine graded cement particles, the water release process from SAP is disturbed and, therefore, prolonged [9]. In a cementitious environment, depending on the water-to-binder ratio of the cement paste and type of cement used for its preparation, SAP reaches its maximal water absorption capacity within minutes [23]. 

Thereafter, due to cement paste being an electrochemically active environment, it desorbs water due to internal stresses within the hardening cement matrix. Due to the lowered amount of water initially absorbed, its water desorption potential is limited both in volume and time. This problem disappears altogether with the change in the dosing method. While initially, by saturating polyacrylic SAP in tap water, in an environment allowing to reach approximately a 10-times higher amount of absorbed water over water absorption in cement paste, water desorption from SAP particles due to the rheology of cement paste is limited. 

Water is desorbed from SAP as an effect of both the electrochemical parameters of the water desorption environment and its physical properties, namely the impact of forming a capillary network in the cement paste. As cement particles in cement paste disturb the flow of water from the SAP to cement matrix, the water desorption rate from SAP slows down, prolonging the hydration time of the matrix and increasing its density over time.

The same mass amount of SAP can absorb different volumes of water (depending on the parameters of the water absorption environment) [25]. In order to include this variable in the concrete design process, a new way of describing the SAP content in a concrete mix, except for the mass criterion, was proposed: the percentage of mixing water that is absorbed by SAP [11]. By dosing non-saturated SAP to the concrete mix, SAP reaches its maximal absorption capacity in a cementitious environment within minutes after introducing it to the mix [11]. On the other hand, in the case of introducing hydrogel, SAP reaches its maximal water absorption capacity in tap water prior to the mixing process [9]. As those two water absorption capacities vary, for the same mass amount of SAP, the percentage of absorbed water during the mixing phase is different.

With changes to the pore network of cementitious composites caused by SAP addition, it is expected that the composite properties will change as well. Most notably, the mechanical performance of such a composite is going to be affected [15,26]. With a change in the pore size distribution and density of the cement matrix, the permeability of a SAP-modified material will also vary from the non-modified variant. SAP is sometimes compared to air-entraining agents, as its effect on the pore network increases the freeze–thaw resistance of cementitious composites [27]. This property of SAP-modified concrete is usually linked with a presence of macropores that limits the continuity of the capillary network of the cementitious material [20].

Any change to the pore network in concrete results in changes in the durability of the material. This can either facilitate the movement of corrosive agents into the matrix of the material or slow it down. As concrete is a construction material meant to last for decades, investigating the influence of admixtures and additions to the concrete mix is essential to study the durability. The main issue with changes to the permeability of SAP-modified concrete is caused by additional water replacing the amount of water stored in the SAP structure. By having an additional volume of water in the composite during the mixing stage, the porosity of the cement matrix is increased, thus, resulting in a deterioration in concrete’s mechanical performance, which usually has a negative effect on its durability [28].

When SAP particles desorb water in a cement matrix, due to changes in pressures within the pore network, its structure is altered—the capillary network loses its continuity. As a result, the SAP-modified concrete is characterized by an increased freeze–thaw resistance [28]. Due to this fact, one can assume that SAP addition will impact the transport properties of other substances through the pore network except for water. However, research attempts to study the diffusion of chloride ions to investigate the issue are sparse. 

The durability of cementitious materials is linked with the susceptibility to aggression by chemical agents from the outer environment. The deterioration of hardened concrete can be caused by many corrosive agents that enter cementitious composites through its pore network. Therefore, altering the pore network distribution by any means of internal curing would change its durability. SAP influences several core properties of the cement matrix, including its density and pore distribution [20]. With its impact on the properties of concrete, it is expected that chloride ion diffusion would also be impacted by SAP addition to the concrete mix.

Methodologically, internal curing by LWA and SAP is similar. Both methods usually include adding additional water to the concrete mix to increase the amount of free water in forming a cement matrix. However, while LWA addition is based on purely mechanical means of carrying water, in internal curing by SAP, this effect is strengthened by the impermanently bound water present in its structure and spread out within the entire volume of the cement matrix and not only to the close distance from LWA grains [29,30]. The main issue with concrete permeability is its dependence on the amount of water in the concrete mix. As this amount grows with the increase of the water-to-binder ratio, so does the permeability of hardened concrete, which would negatively affect the transport properties of corrosive agents through the pore network of the composite.

### 1.3. Research Significance and Aims

The conducted study was designed in such a way as to investigate the influence of the material characteristics of SAP (particle size), dosing method (non-saturated/saturated), and dosing methodology (the addition of extra water to the concrete mix to compensate for water initially stored in SAP) on the chloride ion diffusion in a hardened composite. SAP influences several properties of concrete. 

To correctly assess its impact on ion transport properties through the pore network, the parameters of the aforementioned network had to be designed in a way that allowed comparison with a reference series. It is known that SAP mainly influences two of concrete’s properties—the pore network and cement matrix density. If the balance between those two altered parameters is met, the mechanical properties, namely the compressive strength of SAP-modified concrete, should be similar to the reference series. 

If the amount of added SAP is too high due to either the methodology of dosing (addition of additional water) or the method of dosing (SAP in a non-saturated state tends to form conglomerates within the cement matrix [26]). In that case, its influence over the pore network exceeds its impact on the cement matrix density and results in a deterioration in mechanical properties. By maintaining the balance and knowing that there are changes to the pore network due to SAP addition, this approach to the issue provides ground rules for investigating the influence of SAP on the concrete properties linked with the durability of the material.

## 2. Materials and Methods

The plan of the experiment consisted of two main stages. In the first phase, the water absorption characteristics and particle size distribution of the tested SAPs were investigated. In the second phase, to investigate the influence of SAP only on the properties concerning permeability, its impact on other parameters had to be mitigated. One of the key factors that controls the porosity of the cement matrix is the initial water-to-binder ratio. With that in mind, to choose a proper concrete reference series, preliminary tests concerning SAP influence on the compressive strength were conducted. 

The assumption was that there is a border value for the water-to-binder ratio above which the influence of a given amount of SAP on the mechanical properties is negligible. Knowing that different polyacrylic SAPs differ between themselves in terms of properties, including the impact of aggregate and binder on mechanical properties of concrete, the water-to-binder ratio found in preliminary tests is not an absolute value for which there is no influence of SAP on concrete’s mechanical properties. Instead, it represents the borderline value for materials and SAPs used in the conducted study.

Having found that value of the water-to-binder ratio of 0.4 provided indirect evidence that there is a way to achieve an equilibrium between negative changes, from the mechanical perspective, to the pore network and positive changes—an increase in the cement matrix density. Based on those findings, for a chosen water-to-binder ratio of 0.4 and the percentage amount of mixing water being initially absorbed by SAP (5% for non-saturated SAP and 50% for water-saturated SAP), in the second phase of the experiment, the influence of the polyacrylic SAP material characteristics, dosing method, and dosing methodology on the chloride ion diffusion was investigated. Additionally, to verify the influence of the negligible impact of the given SAP dosage and water-to-cement ratio on the mechanical properties, compressive strength tests were performed on the designed concretes, both modified with SAP and reference series.

### 2.1. Materials

CEM I 42.5 R cement (GÓRAŻDŻE, Poland) used in the conducted research fulfilled the requirements of EN 197 [31]. Mineral fine aggregate (Vistula River sand, Warsaw, Poland) and natural gravel aggregate (2/8 mm) (Warsaw, Poland) used in the conducted research fulfilled the requirements of EN 12620 standard [32]. Tap water used in the conducted research fulfilled the requirements of EN 1008 [33]. In addition, a superplasticizer (CHRYSO, Warsaw, Poland), characterized by a steric and electrostatic mechanism of action and a maximum content allowed by the manufacturer in the concrete mix at the level of 3% of the binder mass, was used. The superplasticizer was added after the introduction of SAP to a concrete mix.

**Figure 1:**
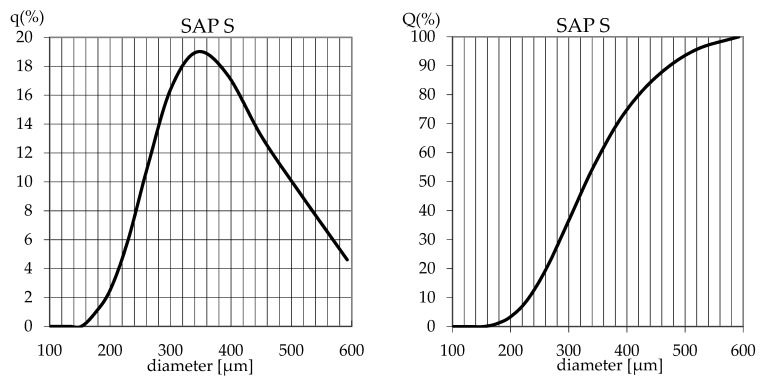
Particle size distribution of SAP S measured through laser diffraction.

**Figure 2:**
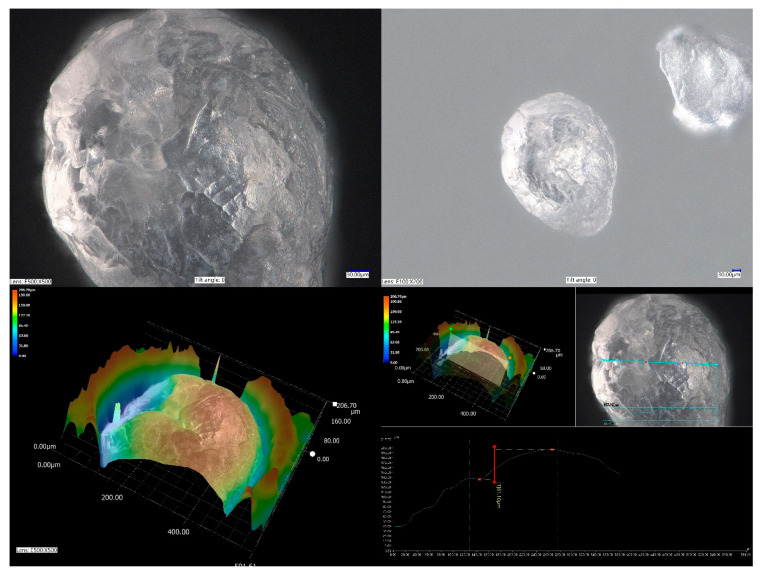
Microscopic photograph of non-saturated SAP S particle in dry conditions.

**Figure 3:**
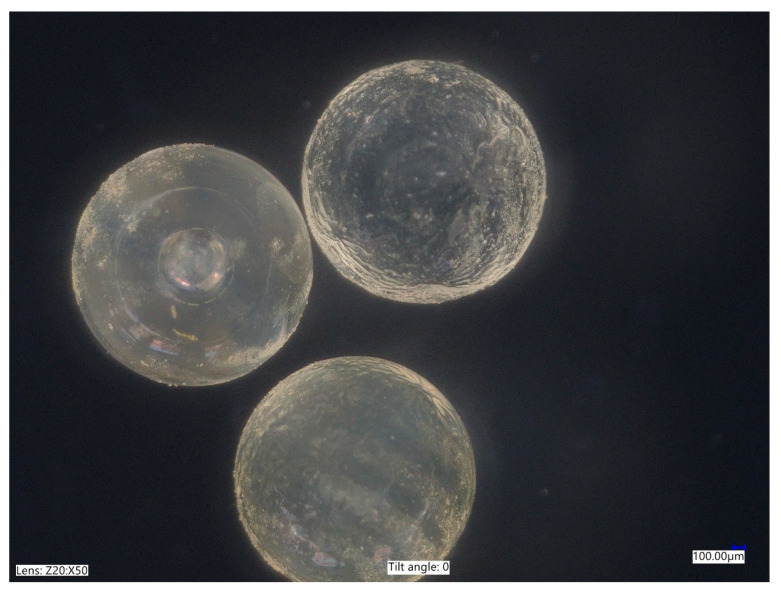
Microscopic photograph of non-saturated SAP B in dry conditions.

Two polyacrylic SAPs with different grain sizes and water absorption properties were used. Polyacrylic SAP S (sodium acrylate and acrylic acid polymer) (BASF, Ludwigshafen, Germany) of particle size distribution presented at Figure 1 characterized by the median grain size of 330 µm, maximal particle size was approximately 600 µm, with a regular, close to spherical grain morphology (Figure 2) and a polyacrylic SAP B (sodium acrylate and 2-propenoic acid polymer) (DEMI CO., LTD, Zhuhai, China), which was characterized by granulation in a dry state of between 2.0 and 2.5 mm and spherical grain morphology (Figure 3).

Two reference series were designed to investigate the influence of SAP on the permeability of concrete. REF 1 of a water-to-cement ratio of 0.4 was a reference series for SAP-modified concretes without any additional water, while REF 2 of water-to-cement ratio of 0.42 was a reference series for SAP-modified concretes with additional water (Table 1). The additional water in the amount of w/c = 0.02 was added to have a reference series for the SAP-modified series with increased total water-to-cement ratio. To all series, both reference and modified with SAP, the same amount of superplasticizer was used, namely 2% m.c.

### 2.2. Methods

Several properties of both SAP and concrete were investigated during this study. All the methods for each of the experiments are presented in this chapter.

#### 2.2.1. SAP Water Absorption Characteristics

To investigate the SAP water absorption characteristics in a cementitious environment, cement pastes that served as water absorption environment for SAP were prepared. Those were characterized by different water-binder ratios (Table 2). The water–cement ratios of the tested cement pastes were designed between 0.4 and 500. The cement paste with a water–cement ratio of 500 simulated a water environment with a pH level of 12.3.

A regular teabag method test was conducted to estimate the influence of the water-binder ratio of the cement paste on the water absorption properties of SAP. This included sealing a known mass of SAP in a teabag of a known mass and measuring the increase in mass dependent on time spent in the water absorption environment. A reference sample with no sealed SAP was prepared and put into cement paste of the same water–binder ratio to mitigate the influence of cement paste gathering on the outer side of the teabag. 

The mass increase on the reference sample was then subtracted from the sample with sealed SAP at every measurement point. The test lasted for 60 min for SAP S as this proved sufficient to reach the maximal water absorption capacity of SAP in each of the tested cement pastes. For SAP D, which required more time to reach the maximal absorption capacity in each environment, the water absorption tests lasted 24 h.

#### 2.2.2. Concrete Mix and Hardened Concrete Test Procedures

The consistency of the concrete mixes was evaluated via slump test according to PN-EN 12350-2 [34]. The compressive strength of hardened concrete was evaluated in accordance with EN 12390-3 [35] standard after 28 days of curing in a curing chamber (RH of 95% and temperature of 22 °C) on cubic samples (150 mm × 150 mm × 150 mm).

The chloride ion diffusion test was carried out on 100 mm × 100 mm × 100 mm samples with total water–cement ratios of 0.4 and 0.42. After demolding, the samples were subjected to a 28-day curing period in a curing chamber (RH of 95% and temperature of 22 °C). After 28 days, the samples were halved and wholly immersed in a 3% NaCl solution. After an additional 30 days, concrete samples were taken from each series at different depths to determine the content of chloride ions. The thickness of each of the taken sample layers was 1 mm. The samples were taken using a specialized profile grinding kit and were stored in a tightly closed string bag.

The chloride ion content was determined using the Volhard method. A sample, previously dried at 105 °C, was weighed in a 250 mL beaker to carry out the study. The weight of a particular sample depended on the thickness of the layer it was taken from.

The weights of individual samples were recorded with an accuracy of 0.001 g. Each of the beakers prepared for the test with the appropriate content of the powdered sample was appropriately described. Each sample was tested by performing the following steps sequentially:

1. Add approximately 50 mL of distilled water to the test sample. Mix the solution thoroughly with a baguette.
2. Add 10 mL of 5 mol/dm^3^ HNO_3_ to the beaker using a pipette. Mix the solution thoroughly with a baguette.
3. Add 50 mL of hot distilled water. Mix the solution thoroughly and covering the beaker afterward with a watch glass.
4. Place the beaker on the burner, and boil of the solution for at least 3 min.
5. Add 5 mL AgNO_3_ at a concentration of 0.05 mol/dm^3^ with a dispenser, mixing the solution thoroughly.
6. Filter the test solution into a conical flask of approximately 250 mL capacity. Rinse the beaker, the funnel, and the filter used to filter the solution with distilled water. The volume of the solution in each flask should be 150–200 mL.
7. Add about 4 mL of indicator to the flask with a solution—a saturated ammonium sulfate (VI) and iron (III) acidified with nitric acid.
8. Titrate with the solution of potassium thiocyanate KSCN with the concentration of 0.05 mol/dm^3^ until the color of the solution changes to a salmon color.
9. Record the reading of the KSCN volume used.

The above steps were also performed for a sample containing no powdered concrete—a ‘blank test’. The result was recorded and used to compile the final results. The formula determined the chloride mass content in individual samples:

(1) $$m_{Cl}=0.035457\cdot \left(V_{AgNo_{3}}\cdot 0.05-V_{KSCN}\cdot C_{KSCN}\right)$$

(2) $$C_{KSCN}=\frac{V_{AgNo_{3}}\cdot C_{AgNO_{3}}}{V_{KSCN}}$$

where $V_{AgNo_{3}}$ is the volume of added AgNO_3_ (5 mL), $V_{KSCN}$ is the volume of added KSCN until reaching the appropriate color of the solution, and $C_{AgNO_{3}}$ equals to 0.05 (from ‘blind test’).

The effective diffusion coefficient of chloride ions was then calculated according to the formula:

(3) $$c=c_{0}\left[1-\mathrm{erf}\left(\frac{x}{2\sqrt{D_{\mathrm{eff}}t}}\right)\right]$$

where *c* is the chloride ion content in concrete at a depth of x, *c*_0_ is the chloride ion content in the near-surface layer of concrete, D_eff_ is the chloride ion diffusion coefficient, and t is the time of diffusion of chloride ions.

#### 2.2.3. Reference and SAP-Modified Series—Plan of the Experiment

The conducted research involved preparing concrete series modified with two types of SAP, which were added to the concrete mix according to three methods—either added in an non-saturated form (D variant), hydrogel form (H variant), or hydrogel form with additional water (HD variant) as shown in Table 3. The amount of extra water added to the concrete mix to compensate for the SAP absorption capacity can be designed based on different approaches to the issue—it could either be based on the properties of the concrete mix to which SAP is added (by adding additional water to the SAP-modified mix until its consistency is comparable with a non-modified variant), or based on the water absorption capacity of the tested SAP. 

In the conducted experiment, the amount of extra water added to the mix in the HD case was calculated as an amount of water that SAP would hold in its structure after the SAP absorption capacity lowered from that of the absorption capacity in tap water to that of the absorption capacity in cement paste of a water-to-binder ratio of 0.4. This approach to the presence of the additional water in the system allowed us to test the influence of the minimal value of extra water to compensate for the water absorbed by SAP. If, even with such a minor modification of w/c_tot,_ the chloride ion diffusion would be affected, all methods, including the addition of a higher volume of water to the mix, would affect it as well.

## 3. Results

### 3.1. SAP Water Absorption Characteristics

**Figure 4:**
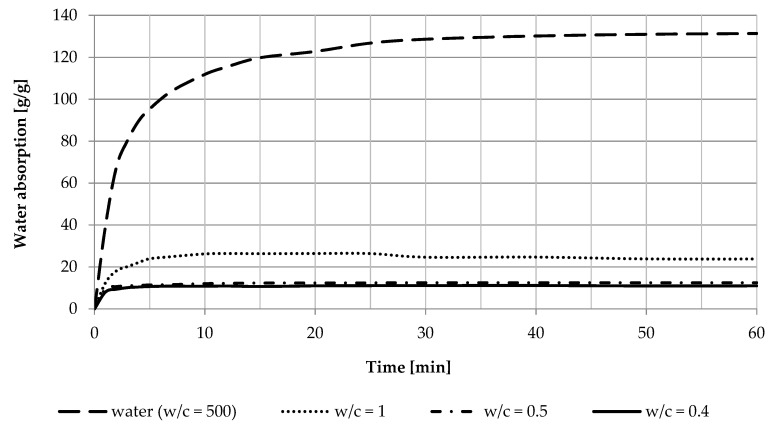
Water absorption capacity as a function of time in water absorption environments of different water-to-cement ratios.

The water absorption capacity in different water absorption environments was studied based on water absorption tests performed on SAP S and SAP B. The absorption of SAP S changed rapidly after introducing SAP S to the water absorption environment (Figure 4). We found that the time required for SAP S to reach its absorption capacity in an environment of different w/c ratio decreased along with the reduction of the w/c ratio. 

While in the solution, which was to simulate tap water environment (w/c = 500), the time required to reach maximal absorption capacity was approximately 40 min. For the environment of the lowest studied w/c = 0.4, it reduced to approximately 2 min. Due to this fact, for concrete series with the addition of non-saturated SAP S, SAP S achieved its maximal water absorption capacity in the cementitious environment during the mixing stage of the concrete mix.

The time required for SAP S to reach its maximal water absorption capacity in any given environment shortened as the water-to-cement ratio of the water absorption environment lowered (Table 4). The SAP S maximal/reference absorption capacity in the environment simulating tap water environment was approximately 135 g/g, while, in the cement paste of 0.4, it reduced to approximately 11 g/g.

SAP B, characterized by a much slower water absorption rate from any environment, was subjected to a water absorption test that lasted for 24 h. Its absorption capacity reduced by approximately 10 times, from approximately 70 g/g in tap water environment to approximately 7 g/g in cement paste of w/c = 0.4 (Table 5). Due to this fact, for concrete series with the addition of non-saturated SAP D, SAP D did not achieve its maximal water absorption capacity in the cementitious environment during the mixing stage of the concrete mix.

### 3.2. Concrete Mix Consistency

**Figure 5:**
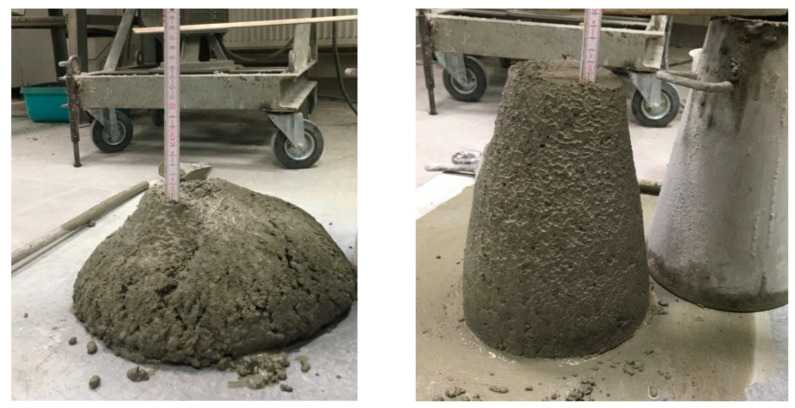
Photograph of the difference in consistency measured with the slump test method between the REF 1 series (**left photo**) and SAP SH50 (**right photo**).

Due to the addition of SAP to the concrete mix, its consistency compared to the reference series was affected (Table 6). While both reference series of w/c_tot_ 0.4 and 0.42 were of consistancy class of S4 measured through slump test (18.0 and 21.0 cm, respectively), every SAP-modified concrete, except for the BD5 series, was characterized with a lower consistency (Figure 5). The series with the lowest flowability was the SAP SH50 series—at 6.5 cm.

### 3.3. Concrete Compressive Strength

After 28 days of curing, compressive strength tests were performed to verify the initial assumption on the negligible effect of SAP S and SAP B on the compressive strength in the designed amount on the concrete with particular total water-to-cement ratio (above 0.4), (Table 7) and with a given amount of mixing water being initially absorbed by SAP. We confirmed that for a selected w/c ratio and aggregate, the influence of the tested SAPs on the compressive strength was negligible.

### 3.4. Concrete Permeability—Chloride Diffusion

Chloride ion diffusion was tested using the Volhard method. As a result of the performed tests, the %Cl^−^ content in 1-mm wide layers for the 1- to 8-mm deep in the concrete samples and chloride ion diffusion coefficients were calculated (Table 8).

## 4. Discussion

Concrete is a material whose properties are linked with the parameters of all phases that it constitutes: the aggregate, cement matrix, and pore network. SAP is one of the least known materials used in concrete technology [20]. The design of SAP-modified concrete is a process that needs to take into account SAP’s influence on both the properties of concrete mix and hardened concrete. 

Its influence can be modified by the type of SAP used, its water absorption and desorption parameters, granulation in the non-saturated state, morphology, method of dosing (non-saturated/hydrogel), or methodology of dosing (with or without extra water) [12]. In the performed research, two polyacrylic superabsorbent polymers were used that differed both in water absorption capacities and particle size distribution in a non-saturated state in order to investigate chloride ion diffusion of SAP-modified concrete, as SAPs influence on ion transport parameters has yet to be fully established [20,36,37].

Internal curing in concrete technology was introduced to positively influence the durability of high-performance concretes [37,38,39]. With an increase in the amount of the binder within the composite, a reduction in the water amount, and an increase in the mass of fine aggregate, the influence of autogenous shrinkage on composite’s properties and durability had to be, among the influence of others, mitigated [20]. As external curing methods proved inadequate to mitigate those phenomena, the issue of modifying the transport of water during and after the hardening of the cement matrix was raised [40]. 

Currently, SAP represents one of the most curious materials used to modify water kinetics in cementitious materials [20] and the hydration process [41]. As presented in the introduction, SAP can be characterized by several material characteristics, including its particle size distribution and water absorption capacity. Both of those parameters need to be taken into account while designing SAP-modified concrete. As non-saturated SAP exists in the form of physical polymer grains, its particle size influences the effect on the parameters of the pore network within the cement matrix. 

In that sense, SAP particle size distribution characteristics need to be taken into account while designing SAP-modified concrete. The other vital parameter of SAP is its water absorption capacity in both tap water (close to the reference water absorption capacity) and in an environment to simulate a cementitious environment. Those two material characteristics are crucial to designing concrete with SAP. With an increase in particle size of SAP in non-saturated form, a negative effect on the mechanical properties of cementitious composites can be observed [42].

Apart from the parameters of SAP, the method and methodology of its dosing into the concrete mix affect its performance in the composite. We propose that differentiation needs to be made concerning the use of SAP and simultaneous addition of extra water to the mix to maintain the modified mix’s consistency on the same level as a reference one. While the workability of any concrete mix is an important issue during the production process [15], maintaining it while modifying concrete with different admixtures and additions usually does not involve additional water in the system. The reason behind such an approach is that any change to the water amount in the mix changes the mechanical performance of hardened concrete. This also influences the durability [43].

Polyacrylic SAPs can absorb different amounts of water in different water absorption environments. In the conducted research, SAP S was characterized by the water absorption capacity in a tap water environment of approximately 135 g/g and in a cementitious environment of approximately 12 g/g. SAP B had a water absorption capacity in the tap water of approximately 70 g/g, while, in a cementitious environment, that value dropped to approximately 7 g/g. The difference in the water absorption capacities allows using SAP as an internal curing agent via different dosing methods. 

Suppose SAP is to be added to a water-containing environment. In that case, SAP grains will absorb water from that medium until they reach their water absorption capacity in that environment. Afterward, that water due to external pressures can be released. To compare two different polyacrylic SAPs, their difference in water absorption capacities had to be taken into account. Therefore, while designing SAP-modified series added in a non-saturated form to the mix, a percentage of absorbed water both by SAP S and SAP B was the comparative parameter. By maintaining it at the same level, due to differences in water absorption capacities between SAP S and SAP B, a different mass amount of each polymer was added to adequate series (in the case of SAP S—0.15% m.c. and in the case of SAP B—0.29% m.c.).

Between the two tested dosing methods (by introducing SAP into the concrete mix either in a non-saturated state or in a hydrogel form), two main differences influence the water desorption process from the SAP structure. The external pressures forcing SAP to release water from its structure, depending on the dosing method, vary. Suppose SAP is introduced in a non-saturated state after reaching its absorption capacity in cementitious environments. In that case, it releases water due to pressures resulting from ongoing hydration and volumetric changes present during hydration. 

Suppose SAP is introduced in a hydrogel form into the concrete mix. In that case, the water desorption process is intensified as SAP is to reduce its initial high water absorption capacity. The intensification in water desorption was found to last approximately 24 h after the introduction of SAP into the concrete mix [9]. For SAP in a hydrogel form, after the absorption of water increases its particle volume—the polymer itself is insoluble. Therefore it maintains a three-dimensional structure. With an extra volume of absorbed water, its network stretches out, making it prone to fragmentation during mixing, resulting in final granulation of the fragmented hydrogel after the desorption of water finer than that in a non-saturated state [9]. 

Not all SAP-based hydrogels are susceptible to such a reduction in particle size. One of the factors controlling the absorption capacity of SAP is the amount of crosslinker is used to hold its structure—the more the crosslinker, the less water SAP can absorb and, in consequence, the less it is susceptible to a reduction in particle size [44]. The introduction of a new variable in the form of the dosing method affected the amount of mixing water being initially absorbed by SAP in hydrogel form. 

As the water absorption capacity of tested polyacrylic SAPs was approximately 10-times higher in a tap water environment than in an appropriate cementitious environment, the amount of initially absorbed mixing water increased approximately 10 times as well. In order to keep the series with different dosing methods comparable between each other, the mass content of SAP in the mix had to be kept at the same level (for SAP S—0.15% m.c. for all SAP S-modified series and 0.29% m.c. for all SAP D-modified series).

In the conducted research, to calculate the influence of both SAP and additional water added to the mix on the chloride ion diffusion, a comparison between all tested series had to be established. As the durability of concrete is of the essence for hardened concrete, not concrete mix, a comparative parameter had to be found between all SAP-modified concretes and reference ones. It was decided by the authors that, by maintaining the compressive strength of all concrete series at the same level, the impact of all discussed variables previously on the chloride ion diffusion could be investigated. 

The prepared reference series (Table 1) and SAP-modified series (Table 3), due to the design process, were characterized by compressive strength at a comparable level (average compressive strength values after 28 days between 49.81 MPa and 53.55 MPa). Different SAP material characteristics [44], SAP dosing methods [9], and methodologies [11] have various effects on the mechanical performance of concrete. These can be positive in some cases (increasing the degree of hydration without compromising pore network parameters [15]) or negative (compromising the pore network by SAP-induced macropores/defects [45,46,47]). Both of these effects had to be mitigated to establish a level field for accurately assessing the impact of all examined SAP-related variables on chloride ion diffusion.

**Figure 6:**
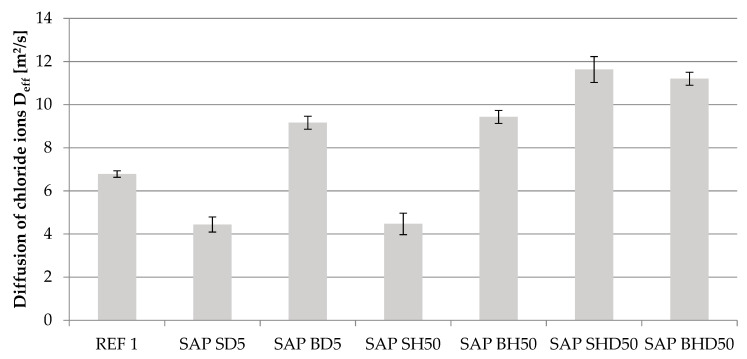
Coefficients of chloride ion diffusion for tested concrete series.

The methodology behind SAP addition to the concrete mix is also relevant when investigating its influence on concrete’s properties [9,11]. From the conducted permeability tests, SAP S reduced the chloride ion diffusion through the pore network for the SAP-modified series, which did not involve additional water being added to the system (Figure 6). 

The chloride diffusion lowered by approximately 30% compared to the REF 1 series of water-to-cement ratio of 0.4 for the SAP S-modified series that did not involve any extra water (from 6.78 × 10^−12^ m^2^/s for REF 1 to 4.44 × 10^−12^ m^2^/s for SAP SD5 and 4.47 × 10^−12^ m^2^/s for SAP SH50). The SAP SH50 series in which w/c_tot_ was increased by 0.02 (the amount of water that was entrained in SAP) resulted in an increase of chloride ion diffusion coefficient by approximately 70% compared to the REF 1 series (an increase from 6.78 × 10^−12^ m^2^/s for the REF 1 series to 11.63 × 10^−12^ m^2^/s for the SAP SHD50 series).

Additional water in the system, even if showing no influence on the mechanical properties of the material (Table 7), impacts the parameters of the cement matrix. The method of increasing w/c_tot_ by the amount of water entrained in SAP led to significant changes in the properties of the cement matrix. Although in the performed tests, the volume of additional water was set to a minimal amount—only to the calculated mass of water that would be absorbed by SAP in a cementitious environment of water-to-cement ratio of 0.4, so that its influence on the mechanical performance would be negligible, it had an impact on the permeability of cement matrix modified in such way. 

The consistency of the tested concrete mixes was also measured (Table 6). We confirmed that the addition of extra water to the concrete mix in order to compensate for the water absorbed by SAP increased the flowability of the concrete mix. By increasing the total water-to-cement ratio by 0.02, the flowability of series changed (from SAP SH50 = 6.5 cm to SAP SHD50 = 15.5 cm under the slump test procedure). However, even with the set additional water in the system, the consistency of reference series was not reached (for the REF 1 series, the consistency was 18.0 cm). It is highly probable that if the method of calculating the entrained amount of water in SAP involved maintaining the consistency of tested concretes on a similar level, its negative influence on the permeability of such concretes would be even greater.

The case of SAP S shows the issue with the popular methodological approach to the design of SAP addition to the concrete mix [11]. Suppose the volume of water entrained in SAP is to be treated as an additional volume of water added to the concrete mix to maintain the effective water-to-cement ratio. In that case, it results in an increase of porosity of cement matrix and, therefore, in an increase of diffusion of corrosive agents. Although if compared to reference series with additional water (REF 2 of water-to-cement ratio of 0.42—Table 8) series modified by SAP S with additional water show the reduction in chloride diffusion, taking a step back to the original reference series shows an increase in permeability of such a concrete by approximately 70%.

**Figure 7:**
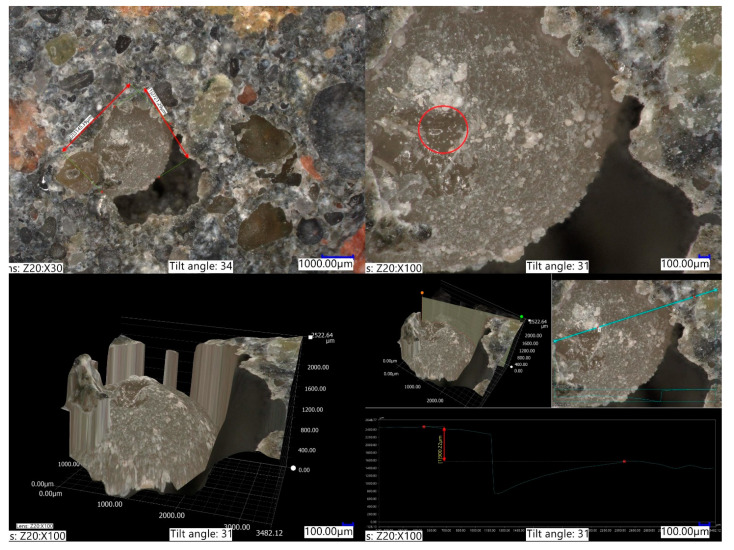
Micrograph of SAP B particle of spherical morphology in SAP BD5 concrete series after exposure to 3% NaCl environment. Red-circled is a fragment of SAP B with a removed outer layer of crystallized NaCl showing a lack of visible salt crystallization within saturated SAP B.

Due to its particle size in non-saturated form and absorption capacity, SAP B indicated a smaller susceptibility to the mechanical reduction of its particles during the mixing period of concrete mix (Figure 7). Due to this fact, its particles had a significant impact on the pore network of tested concretes. The combined effect of large SAP particle size (2–2.5 mm) in a non-saturated state and low water absorption capacity (approximately 70 g/g in tap water environment) contributed to the significant changes in the pore network. 

Based on its properties, it caused an increase in the diffusion of chloride ions compared to the reference series regardless of the dosing method. For the SAP B modified series with no additional water in the system, the diffusion coefficients increased significantly, from 6.78 × 10^−12^ m^2^/s for reference series REF 1 to 9.16 × 10^−12^ m^2^/s for SAP BD5 series and to 9.43 × 10^−12^ m^2^/s for SAP BH50 series. An increase in the chloride ion diffusion was even more significant in the SAP BHD50 series (with additional water in the system), reaching 11.20 × 10^−12^ m^2^/s.

It was shown that, even with control over the mechanical properties of SAP-modified concretes established in the performed research, SAP influenced the chloride ion diffusion. However, to achieve such a setup, the total w/c ratio of the reference series had to be increased to the value of 0.4. In the case of the lower value of the aforementioned ratio, SAPs influence of mechanical performance was more difficult to be contained with the amount of variables that needs to be considered (the SAP material characteristics, the dosing method, and the presence of additional water in the system). 

In the presented study, the authors proposed a method of standardization for testing chloride ion diffusion for both different SAP types and different methods of its introduction into the concrete mix. With SAP being introduced into high-performance concretes of lower w/c, SAPs influence on the chloride ion diffusion would depend on its material characteristics and the presence of additional water in the system. 

Usually, if SAP is added to the concrete mix in a non-saturated state, the mass amount is higher than in the experiment plan proposed by the authors [20,46]. With an increase in its amount, the influence on the consistency of the concrete mix is even greater. If the concrete mix consistency is considered the parameter that is to be maintained at the level of the reference series, the amount of extra water required to compensate for the SAP-entrained one increases. One can speculate that it would further negatively impact the permeability of concrete.

SAP’s influence on the course of hydration of cementitious composites was investigated by measuring its impact on concrete’s different properties—its influence on the development of shrinkage deformations, mechanical strength, freeze–thaw resistance, permeability, hydration, etc. However, the exact phenomena that cause SAP to be an effective internal curing agent are still elusive. In Figure 7, a small fragment of SAP-modified concrete is presented. 

The micrograph was taken on a SAP BR5 sample after 30 days in a 3% NaCl solution. After that time, the sample was fractured, and its internal structure was photographed. It shows an intact SAP B particle, covered in a layer of crystallized NaCl. That level of salt crystallization was only observed on SAP particles, not within the cement matrix. That NaCl layer was partially peeled from the SAP B surface. Its inner volume showed no observable signs of salt presence.

The crystallization process during hydration of cementitious composites is a complex issue that is dependent on a number of variables. We believe that this observation provides indirect evidence of SAP water release mechanism within cement matrix and explains SAPs influence on multiple properties of cementitious materials. Intense crystallization on the surface of SAP indicates a presence of a thin layer of water that is not absorbed by SAP. It acts as an intermediate environment between that of saturated SAP and the outer environment. 

It also serves as a crystallization environment, separate from crystallization not in the vicinity of SAP particles. Due to the low volume of water that it consists of, the convergence point for crystallization, in this case, the appropriate NaCl concentration, is achieved faster than in the rest of the composite, resulting in the crystallization of salt on the surface of polyacrylic SAP. 

Due to the increase in Cl^−^ ions concentration in that outer layer of free water on SAP structure, due to the electrochemical nature of SAP absorption capacity, its absorption capacity decreases—SAP desorbs water and self reduces its size, providing the additional water to its outer layer, which serves as a crystallization environment. One can imagine a similar mechanism taking place during the hydration process of binder in a cementitious environment. This approach to the issue of mechanisms behind SAP influence on the properties of cementitious composites and ultimately on the design of SAP-modified concrete is going to be investigated by the authors in future research.

## 5. Conclusions

In the conducted research, the SAP parameters (granulation and water absorption capacities in different environments), concrete mix consistency, hardened concrete compressive strength after 28 days, and chloride ion diffusion of SAP-modified concrete were investigated. Based on performed tests and experiment plan, the following conclusions can be made:

- The designed experiment plan, with the w/c = 0.4 and the mass amounts of SAP S and SAP B set based on the mass amount required to either absorb 5% or 50% of mixing water (depending on SAP dosing method) allowed for all investigated reference and SAP-modified concretes compressive strength to be kept at a similar level (from compressive strength tests performed after 28 days from sample preparation between 49.81 and 53.55 MPa).
- The initial parameters of SAP—its granulation and water absorption capacity in different environments—influences its impact on the permeability of SAP-modified concrete. Polyacrylic SAP B of 2.0–2.5 mm particle sizes in a non-saturated state, regardless of the dosing method or presence of additional water in the system, increased the permeability of concrete by increasing the chloride ion diffusion by more than 50% compared to the reference series. Polyacrylic SAP S of finer granulation (with median particle size in the non-saturated state of 330 µm, in the cases when it was added to the concrete mix without additional water reduced the chloride ion diffusion by approximately 35% compared to the reference series.
- The increase of w/c_tot_ by the amount of water entrained in SAP contributes to the increase in the permeability of concrete and, therefore, negatively affects its durability. For two investigated polyacrylic SAP, the chloride ion diffusion coefficient increased compared to reference series if additional water in the amount of 0.02 w_e_/c was added to the system—for SAP S, it increased from 6.78 × 10^−12^ m^2^/s for the REF 1 series to 11.63 × 10^−12^ m^2^/s; and, for SAP B, it increased from 6.78 × 10^−12^ m^2^/s for the REF 1 series to 11.20 × 10^−12^ m^2^/s.
- The method of dosing SAP to the concrete mix (non-saturated or in hydrogel form), under the proposed experiment setup, had a negligible impact on the chloride ion diffusion. For the SAP S series—SAP SD5 = 4.44 × 10^−12^ m^2^/s and SAP SH50 = 4.47 × 10^−12^ m^2^/s; and for the SAP B series—SAP BD5 = 9.16 × 10^−12^ m^2^/s and SAP BH50 = 9.43 × 10^−12^ m^2^/s.
- The additional water in the system in the amount of w_e_/c = 0.02 for the series SAP SHD50 influenced its concrete mix consistency. Compared to the variant with no added extra water (SAP HD50), the flowability of concrete mix measured by the slump test increased—from 6.5 cm for the SAP SH50 series to 15.5 cm for the SAP SHD50 series.

The conducted study indicates the premise of the mechanism of the release of water from SAP in cementitious composites, which will be investigated further by the authors in future research.

## Figures and Tables

**Table 1:** Composition of the reference series.

Component of Concrete Mix	REF 1	REF 2
	Amount [kg/m^3^]	Amount [kg/m^3^]
Vistula river sand 0/2	891	882
Gravel 2/8	891	882
Cement CEM I 42.5 R	450	450
Water	180	189
Superplasticizer	9	9
Water–cement ratio [-]	0.4	0.42

**Table 2:** Water-to-cement ratios of cement pastes that served as water absorption/desorption environments.

Cement Paste ID	Water/Cement Ratio [-]
W500	500
CP1	1.0
CP05	0.5
CP04	0.4

**Table 3:** List of prepared reference and SAP-modified concrete mixes.

Series ID	w/c_tot_ [-]	w/c_eff_ [-]	w/c_SAP_ [-]	Superabsorbent Polymer	Dosing Method	Approximately Amount of Mixing Water Absorbed by SAP [%]	Mass of SAP [m.c. %]
REF 1	0.40	0.40	-	-	-	-	-
REF 2	0.42	0.42	-	-	-	-	-
SAP SD5	0.40	0.38	0.02	S	D	5.0	0.15
SAP SH50	0.40	0.20	0.20	S	H	50.0	0.15
SAP SHD50	0.42	0.22	0.20	S	HD	50.0	0.15
SAP BD5	0.40	0.38	0.02	B	D	5.0	0.29
SAP BH50	0.40	0.20	0.20	B	H	50.0	0.29
SAP BHD50	0.42	0.22	0.20	B	HD	50.0	0.29

D—SAP added in an unsaturated form, H—SAP added in a hydrogel form, HD—SAP added in a hydrogel form with additional water.

**Table 4:** The maximal water absorption capacity of SAP S in water absorption environments characterized by different water-to-cement ratios.

Cement Paste ID	Water/Cement Ratio [-]	SAP Water Absorption Capacity [g/g]
*SW500*	500	135.3
*SCP1*	1.0	25.5
*SCP05*	0.5	12.2
*SCP04*	0.4	11.4

**Table 5:** The water absorption capacity of SAP B in water absorption environments characterized by different water-to-cement ratios.

Cement Paste ID	Water/Cement Ratio [-]	SAP Water Absorption Capacity after 24 h [g/g]
DW500	500	70.5
DCP1	1.0	14.9
DCP05	0.5	8.1
DCP04	0.4	7.1

**Table 6:** Consistency of tested concrete series measured by the slump test method.

Series ID	Consistency [cm]	Class
REF 1	18.0	S4
REF 2	21.0	S4
SAP SD5	12.5	S3
SAP SH50	6.5	S2
SAP SHD50	15.5	S3
SAP BD5	20.0	S4
SAP BH50	11.0	S3
SAP BHD50	11.5	S3

**Table 7:** The average compressive strength of SAP-modified and reference concrete series tested after 28 days of curing.

Series ID	Average Compressive Strength after 28 Days [MPa]	Coefficient of Variation [%]
REF 1	53.19	3.25
REF 2	49.53	1.72
SAP SD5	53.69	0.99
SAP SH50	52.49	2.80
SAP SHD50	49.81	4.08
SAP BD5	53.55	2.21
SAP BH50	52.83	3.52
SAP BHD50	50.93	4.05

**Table 8:** The chloride ion diffusion coefficient and the percentage chloride ion content in each 1-mm wide layer of tested concrete series.

Depth [mm]	%Cl^−^ Content (after 30 Days of Cl^−^ Penetration in a 3% NaCl Solution)							
	REF 1	REF 2	SAP SD5	SAP SH50	SAP SHD50	SAP BD5	SAP BH50	SAP BHD50
1–2	0.24	0.20	0.34	0.32	0.28	0.27	0.30	0.30
2–3	0.21	0.20	0.24	0.23	0.23	0.23	0.25	0.26
3–4	0.17	0.18	0.16	0.16	0.21	0.21	0.23	0.24
4–5	0.14	0.17	0.14	0.13	0.19	0.17	0.16	0.20
5–6	0.11	0.15	0.11	0.13	0.19	0.15	0.15	0.17
6–7	0.08	0.14	0.09	0.10	0.14	0.12	0.13	0.15
7–8	0.06	0.12	0.08	0.07	0.11	0.09	0.10	0.12
D_eff_ [10^−12^ m^2^/s]	6.78	22.81	4.44	4.47	11.63	9.16	9.43	11.20

## Data Availability

The data presented in this study are available on request from the corresponding author.
